# Higher Rate of Lymphedema with Inguinal versus Axillary Complete Lymph Node Dissection for Melanoma: A Potential Target for Immediate Lymphatic Reconstruction?

**DOI:** 10.3390/curroncol29080446

**Published:** 2022-08-11

**Authors:** Melina Deban, Patrick Vallance, Evan Jost, J. Gregory McKinnon, Claire Temple-Oberle

**Affiliations:** 1 Surgical Oncology, Tom Baker Cancer Center, Calgary, AB T2N 4N2, Canada; 2 Plastic and Reconstructive Surgery, Tom Baker Cancer Center, Calgary, AB T2N 4N2, Canada

**Keywords:** melanoma, lymphedema, lymph node dissection, lymphatic reconstruction

## Abstract

Background: The present study was conducted to define the lymphedema rate at our institution in patients undergoing axillary (ALND) or inguinal (ILND) lymph node dissection (LND) for melanoma. It aimed to examine risk factors predisposing patients to a higher rate of lymphedema, highlighting which patients could be targeted for immediate lymphatic reconstruction (ILR). Methods: A retrospective chart review was conducted between October 2015 and July 2020 to identify patients who had undergone ALND or ILND for melanoma. The main outcome measures were rates of transient and permanent lymphedema. Univariate and multivariate analyses were performed to assess the relationship between lymphedema rate and factors related to patient characteristics, surgical procedure, pathology findings, and adjuvant treatment. Results: Between October 2015 and July 2020, 66 patients underwent LND for melanoma: 34 patients underwent ALND and 32 patients underwent ILND. At a median follow-up of 29 months, 85.3% (*n* = 29) of patients having had an ALND did not experience lymphedema, versus 50.0% (*n* = 16) of ILND (*p* = 0.0019). The rates of permanent lymphedema for patients having undergone ALND and ILND were 11.8% (*n* = 4) and 37.5% (*n* = 12) respectively (*p* = 0.016, NS). The rate of transient lymphedema was 2.9% (*n* = 1) for ALND and 12.5% (*n* = 4) for ILND (*p* = 0.13, NS). On univariate analysis, the location of LND and wound infection were found to be significant factors for lymphedema. On multivariate analysis, only the location of LND remained a significant predictor, with the inguinal location predisposing to lymphedema. Conclusion: This study highlights the high rate of lymphedema following ILND for melanoma and is a potential target for future patients to be considered for ILR.

## 1. Introduction

Complete axillary (ALND) or inguinal (ILND) lymph node dissection in melanoma carries significant morbidity related to wound healing and lymphedema. This can translate to a worsened quality of life and limb function. Compared with sentinel lymph node biopsy, complete lymph node dissection results in diminished physical functioning, inability to carry out usual roles, fatigue, and pain [1]. This has steered leaders in the field to examine the role of active surveillance and demonstrate that completion lymph node dissection can be supplanted by ultrasound surveillance, limiting the role of lymph node dissection to the therapeutic setting. In the landmark trial MSLT-II, completion lymph node dissection did not increase melanoma-specific survival compared with active surveillance in patients with sentinel-lymph node metastases. [2]. Nonetheless, therapeutic lymph node dissection (TLND) remains a part of the surgical management of some melanoma patients, such as those with bulky nodal disease and those who recur in the nodal basin on active surveillance protocols after a positive SLN biopsy.

Lymphedema has many definitions, and none has been uniformly agreed upon. Pathophysiologically, it is thought to represent an accumulation of protein-rich fluid in the interstitial space, which can be followed by inflammation and fibrosis [3,4]. Clinically, it can be defined as a difference in circumference of 2 cm or more between the affected and contralateral limbs or a volume difference of 10% between limbs [5,6]. Other recent definitions incorporating changes in volume and circumference of the ipsilateral limb over time have been described [7,8,9,10], as well as bioimpedance spectroscopy, perometry [11], and patient reports [12,13,14,15]. This may explain the wide range of lymphedema prevalence, reported between 15% and 62% [2,7,16,17]. However, surgical technique and extent of the dissection, its anatomic location, and the use of adjuvant radiotherapy to the nodal basin may also account for this difference. Risk factors that have been shown to contribute to lymphedema include use of radiotherapy, number of lymph nodes removed, and pathologic presence of disease, obesity, chronic venous insufficiency, and peripheral vascular disease [7,16,18].

In order to mitigate the risk of lymphedema following lymph node dissection, a novel technique of immediate lymphatic reconstruction (ILR) was introduced by Boccardo and colleagues in 2009 [19]. They described a microsurgical procedure where blue dye was injected to identify lymphatics at the time of axillary dissection, allowing preservation of these lymphatics to be later anastomosed to a vein (lymphaticovenous anastomoses (LVA)). In this initial series, in 18 of 19 patients, suitable lymphatics and veins were preserved and anastomosed. At one year of follow-up, no patient had developed lymphedema.

The present study was conducted to define the lymphedema rate at our institution in patients undergoing axillary or inguinal lymph node dissection for melanoma. It also aimed to examine potential risk factors predisposing patients to a higher rate of lymphedema, highlighting which patients could be triaged to ILR.

## 2. Methods

After Institutional research ethics board approval was obtained (HREBA.CC-20-0465), a retrospective chart review was performed of patients at the Tom Baker Cancer Centre in Calgary, AB, Canada. Patients with a diagnosis of melanoma and undergoing a complete lymph node dissection of the axilla or groin were identified using the Synoptec© (Softworks Group Inc., Edmonton, AB, Canada, 2014) database in Calgary between 1 October 2015, and 31 July 2020.

Patients with melanoma were included if they were older than 18 and underwent either an axillary or inguinal lymph node dissection. Patients were excluded if they had distant metastatic disease or concomitant cancers, or if there was evidence of another etiology for their edema (e.g., liver disease, chronic malnourishment, or dialysis).

The primary outcome was the development of lymphedema and whether it was transient with complete resolution without treatment, or permanent. The variables of interest were extracted from patient paper charts and the electronic medical record using a pre-defined collection form. Lymphedema was defined as clinician-reported arm or leg swelling. Permanent lymphedema was defined as lymphedema at the last documented follow-up. Transient lymphedema was defined as lymphedema that resolved during clinical follow-up.

Other variables included were patient factors (e.g., the presence of cardiovascular disease, or neurologic compromise), surgical procedure details (e.g., location and level of lymph node dissection; ALND (level I and II or I, II, and III) and ILND (inguinal or ilioinguinal)), characteristics of the surgical pathology (e.g., location and depth of primary melanoma), nodal burden within the pathological lymph node specimen (e.g., size of largest nodal deposit, number of nodes involved, and presence of extra-nodal extension), and treatment factors related to adjuvant therapy (e.g., radiation, chemotherapy, and immunotherapy). Where lymphedema was identified, the type of supportive care (e.g., compression stockings, therapist referral, or other support) was also gathered.

All data visualization and statistical analyses were performed using Python (open-source) programming language and IBM SPSS, version 26 (Chicago, IL, USA). Descriptive statistics were conducted to provide summary statistics of numerical variables. Various plots aimed to better understand the relationship between different features with outcome.

Univariate analysis was initially performed to find significant predictors of lymphedema, which informed the variables for the logistic regression. Chi-square statistical test or Fisher’s exact test were used to compare categorical variables with outcome. The *p*-values of less than 0.05 were considered significant. For comparison of lymphedema (none, transient, permanent), a *p*-value less than 0.008 was considered significant after Bonferroni adjustment.

## 3. Results

### 3.1. Patient and Tumour Characteristics

From October 2015 to July 2020, sixty-six patients were identified with a diagnosis of lymph-node positive melanoma and who underwent either ALND (*n* = 34, 52%) or ILND (*n* = 32, 48%). In the overall population, the average age of patients was 60 years (26–86 years) with a predominance of male sex (*n* = 42). The average Breslow depth of the primary lesion was 4.4 mm (0.5–38 mm). The only number of lymph nodes resected differed between ALND and ILND (27 vs. 14, *p* < 0.01). There was no difference in age, gender, Breslow thickness of primary melanoma, level of lymph node dissection, therapeutic lymph node dissection, size of largest nodal tumor deposit, presence of extranodal extension, use of adjuvant radiation, and length of follow-up (Table 1).

### 3.2. Extent of Dissection

In terms of the extent of surgical lymph node dissection, 27 of the 34 ALND patients (79.4%) had all three levels cleared. 24 of the 32 ILND (75.0%) had iliac and obturator dissection in addition to the inguinal dissection. Of the patients undergoing ALND, 26 patients (76.5%) were for therapeutic indications for gross bulky disease, versus 21 patients (65.6%) for ILND. The remainder had completed lymph node dissections following positive sentinel node biopsies. The mean number of lymph nodes resected was higher for ALND than ILND (27 vs. 14, *p* = 0.01) (Table 1).

### 3.3. Adjuvant Therapy

Regarding adjuvant therapy, 19 (29%) patients underwent radiation of the nodal basin (nine patients with ALND and ten patients with ILND). Forty patients (61%) received adjuvant immunotherapy and five patients (8%) had adjuvant targeted therapy (Table 1).

### 3.4. Complications and Lymphedema

The rate of wound infection was lower for ALND compared to ILND (20.6% vs. 46.9%, *p* = 0.02). Table 2 reports post-operative outcomes in axillary and inguinal lymph node dissections.

The overall rate of permanent lymphedema was 24.2% (16/66) at a median follow-up of 29 months (3–128 months). 85.3% (*n* = 29) of patients having undergone ALND did not experience lymphedema, versus 50.0% (*n* = 16) of patients having undergone ILND (*p* = 0.0019). The rates of transient and permanent lymphedema were 2.9% (*n* = 1) and 11.8% (*n* = 4) for ALND, respectively. In contrast, for ILND, the rates of transient and permanent lymphedema were 12.5% (*n* = 4) and 37.5% (*n* = 12). A post-hoc chi-square analysis was performed to see which levels of lymphedema differed between ALND and ILND (Bonferroni adjusted at alpha = 0.0083). The difference in transient and permanent lymphedema between ALND and ILND was not significant, but the absence of lymphedema was (Table 2).

### 3.5. Univariate Comparisons

Univariate analysis revealed that the rate of lymphedema was significantly increased if the patient underwent an inguinal lymph node dissection (OR 5.80, 95% CI 1.79–18.78) or if the patient had a post-operative wound infection (OR 3.40, 95% CI 1.14–10.15). Through univariate analysis, there was a signal, though not significant, that the number of nodes resected, extent of nodal resection, and adjuvant radiation may influence the rate of lymphedema (Table 3).

### 3.6. Multivariate Analysis

Multivariate analysis included the following predictors: therapeutic vs. completion lymph node dissection, wound infection, level of lymph node dissection, location of lymph node dissection, and use of adjuvant radiation. The only significant variable for lymphedema was the location of lymph node dissection (inguinal vs. axillary OR 5.37, 95% CI 1.47–19.56). TLND vs. CLND, adjuvant radiation, wound infection, and level of nodal resection did not reach statistical significance for influence on lymphedema rates (Table 4).

### 3.7. Supportive Therapy

Use of supportive care for patients with lymphedema is described in Table 5. Out of the five patients who had undergone an ALND who experienced transient or permanent lymphedema, two were referred to a therapist specializing in conservative treatment of lymphedema, and one was referred to a therapist and prescribed a compression stocking. For patients having undergone an ILND, two patients were prescribed a compression stocking, one was referred to a therapist, and nine received both treatment modalities. The remaining patients had no documentation of treatment for lymphedema.

## 4. Discussion

Lymphedema is a burdensome consequence of lymph node dissection in many patients, resulting in a decline in quality of life. It has been widely shown to increase economic [20], psychosocial, emotional, physical, and relational distress [1,21]. Patients experiencing lymphedema report negative feelings of anxiety, frustration, fear, and increased self-consciousness [21,22]. This has led to a collective effort to decrease the burden of lymphedema by trying to avoid LND when possible, opting for ultrasound surveillance for positive nodes, and in cases where the procedure cannot be avoided, such as in those presenting with bulky disease or those progressing on US surveillance, adding in ILR to try and re-establish lymphatic flow to the venous system from the limb at risk. [19]. As we gained experience in ILR, we aimed to identify risk factors that could assist in selecting patients who were most likely to benefit from this technique.

### 4.1. High Rates of Lymphedema

In our institution, the rate of permanent lymphedema was 11.8% for ALND and 37.5% for ILND, roughly three-fold. The retrospective nature of our study likely understates this rate. This compares similarly to previously reported rates in various studies, ranging from 15 to 62% [2,7,16,17]. In MSLT-1, the rate of lymphedema was 24.1% [2], when axillary and inguinal dissections were grouped together. This is almost identical to our permanent lymphedema overall rate of 24.2%.

### 4.2. Importance of Distinguishing Location of Lymph Node Dissection

Grouping axillary and inguinal dissections together is a suboptimal representation of the risk of lymphedema. As demonstrated in this study, these dissections represent two different entities. The absolute difference in the rate of lymphedema between ALND and ILND was estimated at 25.7%. The location of lymph node dissection is the only factor shown to be associated with lymphedema on both univariate and multivariate analysis.

Other groups have also reported similarly high lymphedema rates for ILND [16,23,24,25]. Cormier and colleagues [16] pooled results from six melanoma studies and showed a lymphedema rate of 18% for ILND and 3% for ALND. Though the absolute numbers may be difficult to compare to our current study given the possible heterogeneity in patient population and lymphedema definition, the relatively higher rate of lymphedema in ILND compared to ALND is a common finding across various studies. It was recently confirmed by another study [24] showing an OR of 6.91 on multivariate analysis for axillary vs. inguinal lymph node surgery.

### 4.3. Lymphedema and Post-Operative Complications

ILND confers a higher risk of lymphedema but also up to a ten-fold risk of overall post-operative complications [23]. Serpell et al. [26] showed a higher rate of wound-related morbidity with ILND compared to ALND. Regarding delayed healing and seroma, ALND compared favorably to ILND. The odds ratio for overall wound complications of ILND vs. ALND was 3.663 on multivariate analysis. These findings further reinforce the concept that dissection location, namely inguinal dissection, is a factor for poor healing and outcomes.

In the same study [26], wound infection rates were approximately 10% lower than ours. Their lower rate of infection may be explained by the extended use of antibiotics postoperatively or their strict definition of wound infection. In contrast, our study relied on clinician evaluation, documentation, and prescription of antibiotics. A common point between both findings is the higher rate of wound infection for ILND. It is unclear if this translates into an increased rate of lymphedema.

In the present study, on univariate analysis, wound infection was one of the factors found to be related to lymphedema. The association was lost on multivariate analysis. Similarly, in a study recently published by Gjorup et al. [24], postoperative infection conferred an OR of 4.39 for lymphedema on univariate analysis, but the association was not observed on multivariate analysis. The relationship between lymphedema and post-operative complications, among which infection, is unclear at this point in time, and causality cannot be established with certainty. A plausible hypothesis is that severed lymphatics pour nutrient-rich contents into the surgical site and provide a favorable environment for bacteria to grow in. Inversely, one could imply that the presence of infection and its treatment (wound debridement and packing) further damages the lymphatics and contributes to the genesis of lymphedema. To our knowledge, none of these hypotheses have been confirmed in the literature.

### 4.4. Radiation Was Not Predictive of Lymphedema in the Present Series

Our data did not find an association between radiation and lymphedema. This is in contradiction with studies showing an increased rate of lymphedema with the combination of surgery and radiotherapy compared with surgery alone [7,16]. We cannot establish with certainty why no link was found between radiation and lymphedema in our study. In a study in which 42% of patients had ILND for a diagnosis of melanoma, radiation was found to be a nonsignificant univariate predictor of lymphedema, in keeping with the current findings [18]. The technique and timing of radiation could be potential risk factors and should be explored in future studies.

### 4.5. Completion versus Therapeutic Lymph Node Dissection and the Impact on Lymphedema

Finally, CLND vs. TLND neared but did not reach statistical significance in our multivariate analysis. This echoes findings from Moody et al. [17], who showed higher but statistically non-significant rates of lymphedema, wound morbidity, and overall complications for TLND.

### 4.6. Limitations

Our study is limited by the number of patients—the collection of data began in 2015, when fewer patients with positive sentinel node biopsies underwent CLND and were managed with ultrasound surveillance. The caveat is that with declining CLND rates, TLND, with more bulky disease, was more common. This patient cohort reflects the current era, where the number of patients undergoing CLND is expected to be smaller. The study is furthermore limited by its retrospective nature and relies on clinician documentation of complications. Similar to every study in the field of lymphedema, we have also been confronted with variable definitions of this condition and have had to ultimately rely on physician evaluation and documentation.

### 4.7. Future Directions

ILR has a budding bright future, with systematic reviews showing a potential benefit in the prophylactic setting [27]. It has been reported in the specific population of patients with locoregionally advanced melanoma, with encouraging feasibility data [28]. A small study of 7 patients with lower extremity skin cancer raised the concern of spreading metastasis via the lymphovenous route, resulting in lower overall survival [29]. However, this is not consistent with the pathophysiology of metastatic spread in melanoma. Reticker-Flynn et al. have recently demonstrated a “Metastatic Tolerance” model, where lymph node colonization by tumor cells induces an immune tolerance, facilitating distant metastasis [30]. When lympho-systemic spread and tumor-immune tolerance have already occurred, it is not plausible that ILR creates a new channel for the spread of tumor cells that are located distally. Moreover, if this theory were accurate, patients with lymphedema would have fewer distant metastases than patients without. To our knowledge, no such report exists. As mentioned by Cakmakoglu et al. [28], in patients with bulky disease where melanoma is considered a systemic disease, ILR is intended to improve quality of life and not oncological outcomes. The latter goal is achieved with adjuvant therapy, which the vast majority of patients receive. The question of the oncological safety of ILR is best answered with a prospective study design, and it will be addressed in the randomized controlled trial LYMbR (Prophylactic LYMphatic Reconstruction to Prevent Lymphedema After Node Dissection for Cutaneous Malignancies; NCT05136079).

## 5. Conclusions

Our institution reports an overall permanent lymphedema rate of 24.2%, with higher rates observed in ILND compared with ALND. Location of lymph node dissection and wound infection were associated with lymphedema on univariate analysis, but only location of lymph node dissection was associated with lymphedema on multivariate analysis. This highlights the high rate of lymphedema for ILND and is a potential target for future improvement using ILR.

## Figures and Tables

**Table 1:** Patient Demographics and Treatment Characteristics.

	ALND (*n* = 34) Mean (Min–Max) or N (%)	ILND (*n* = 32) Mean (Min–Max) or N (%)	*p*-Value
Age (years)	63 (26–86)	56 (28–84)	0.07
Gender (M)	24 (70.6%)	18 (56.3%)	0.23
Breslow thickness of primary melanoma (mm)	5.1 (0.7–38)	3.8 (0.5–8.4)	0.40
Level of LND (level III or ilioinguinal)	27 (79.4%)	24 (75.0%)	0.67
Therapeutic (vs. completion) LND	26 (76.5%)	21 (65.6%)	0.33
Number of lymph nodes resected	27 (10–59)	14 (4–29)	<0.01
Size of largest nodal tumor deposit (mm)	31 (0–80)	19 (0–81)	0.09
Presence of extranodal extension	17 (50.0%)	10 (31.3%)	0.15
Adjuvant radiation (%)	9 (26.5%)	10 (31.3%)	0.72
Length of follow-up (months)	25 (3–86)	34 (3–128)	0.11

**Table 2:** Post-operative outcomes in axillary and inguinal lymph node dissections.

	ALND (*n* = 34)	ILND (*n* = 32)	*p*-Value
No lymphedema	29 (85.3%)	16 (50.0%)	0.0019 *
Transient lymphedema	1 (2.9%)	4 (12.5%)	0.0164 *
Permanent lymphedema	4 (11.8%)	12 (37.5%)	0.1336 *
Wound infection	7 (20.6%)	15 (46.9%)	0.024

* Bonferroni adjusted at alpha = 0.0083.

**Table 3:** Univariate analysis of the rate of lymphedema compared with predictive factors.

Variable	Odds Ratio (95% Confidence Interval)
Age	0.98 (0.95–1.02)
Gender (M vs. F)	0.90 (0.31–2.62)
Breslow of primary melanoma	1.03 (0.93–1.14)
Number of lymph nodes resected	0.97 (0.93–1.02)
Size of largest tumor nodal deposit	1.00 (0.97–1.02)
Presence of extranodal extension	0.95 (0.32–2.77)
Level III/ilioinguinal level of dissection	1.38 (0.38–4.97)
Location of lymph node dissection (inguinal versus axillary)	5.80 (1.79–18.78)
Adjuvant radiation	0.64 (0.18–2.25)
Previous sentinel lymph node biopsy	1.86 (0.65–5.35)
Therapeutic vs. completion lymph node dissection	0.38 (0.13–1.16)
Wound infection	3.40 (1.14–10.15)

**Table 4:** Multivariate analysis of the rate of lymphedema compared with predictive factors.

Variable	Odds Ratio (95% Confidence Interval)
Therapeutic vs. completion lymph node dissection	4.14 (0.93–18.42)
Wound infection	2.58 (0.73–9.15)
Level III/ilioinguinal level of dissection	3.23 (0.57–18.36)
Location of lymph node dissection (inguinal versus axillary)	5.37 (1.47–19.56)
Adjuvant radiation	1.30 (0.30–5.70)

**Table 5:** Use of supportive care for patients with lymphedema.

	ALND (*n* = 5)	ILND (*n* = 16)
Compression stocking	0 (0%)	2 (12.5%)
Therapist	2 (40.0%)	1 (6.3%)
Both	1 (20.0%)	9 (56.3%)

## Data Availability

The data presented in this study is available on request from the corresponding author.

## References

[1] de Vries M., Hoekstra H.J., Hoekstra-Weebers J.E. (2009). Quality of life after axillary or groin sentinel lymph node biopsy, with or without completion lymph node dissection, in patients with cutaneous melanoma. Ann. Surg. Oncol..

[2] Faries M.B., Thompson J.F., Cochran A.J., Andtbacka R.H., Mozzillo N., Zager J.S., Jahkola T., Bowles T.L., Testori A., Beitsch P.D. (2017). Completion Dissection or Observation for Sentinel-Node Metastasis in Melanoma. N. Engl. J. Med..

[3] de Sire A., Losco L., Lippi L., Spadoni D., Kaciulyte J., Sert G., Ciamarra P., Marcasciano M., Cuomo R., Bolletta A. (2022). Surgical Treatment and Rehabilitation Strategies for Upper and Lower Extremity Lymphedema: A Comprehensive Review. Medicina.

[4] Warren A.G., Brorson H., Borud L.J., Slavin S.A. (2007). Lymphedema: A comprehensive review. Ann. Plast. Surg..

[5] Armer J.M., Stewart B.R. (2005). A comparison of four diagnostic criteria for lymphedema in a post-breast cancer population. Lymphat Res. Biol..

[6] Executive C. (2016). The Diagnosis and Treatment of Peripheral Lymphedema: 2016 Consensus Document of the International Society of Lymphology. Lymphology.

[7] Spillane A.J., Saw R.P., Tucker M., Byth K., Thompson J.F. (2008). Defining lower limb lymphedema after inguinal or ilio-inguinal dissection in patients with melanoma using classification and regression tree analysis. Ann. Surg..

[8] Starritt E.C., Joseph D., McKinnon J.G., Lo S.K., de Wilt J.H., Thompson J.F. (2004). Lymphedema after complete axillary node dissection for melanoma: Assessment using a new, objective definition. Ann. Surg..

[9] Lawenda B.D., Mondry T.E., Johnstone P.A. (2009). Lymphedema: A primer on the identification and management of a chronic condition in oncologic treatment. CA Cancer J. Clin..

[10] Levenhagen K., Davies C., Perdomo M., Ryans K., Gilchrist L. (2017). Diagnosis of Upper Quadrant Lymphedema Secondary to Cancer: Clinical Practice Guideline from the Oncology Section of the American Physical Therapy Association. Phys. Ther..

[11] Hidding J.T., Viehoff P.B., Beurskens C.H., van Laarhoven H.W., Nijhuis-van der Sanden M.W., van der Wees P.J. (2016). Measurement Properties of Instruments for Measuring of Lymphedema: Systematic Review. Phys. Ther..

[12] Augustin M., Conde Montero E., Hagenstrom K., Herberger K., Blome C. (2018). Validation of a short-form of the Freiburg Life Quality Assessment for lymphoedema (FLQA-LS) instrument. Br. J. Dermatol..

[13] Devoogdt N., Van Kampen M., Geraerts I., Coremans T., Christiaens M.R. (2011). Lymphoedema Functioning, Disability and Health questionnaire (Lymph-ICF): Reliability and validity. Phys. Ther..

[14] Klernas P., Johnsson A., Horstmann V., Kristjanson L.J., Johansson K. (2015). Lymphedema Quality of Life Inventory (LyQLI)-Development and investigation of validity and reliability. Qual. Life Res..

[15] Monticone M., Ferriero G., Keeley V., Brunati R., Liquori V., Maggioni S., Restelli M., Giordano A., Franchignoni F. (2021). Lymphedema quality of life questionnaire (LYMQOL): Cross-cultural adaptation and validation in Italian women with upper limb lymphedema after breast cancer. Disabil. Rehabil..

[16] Cormier J.N., Askew R.L., Mungovan K.S., Xing Y., Ross M.I., Armer J.M. (2010). Lymphedema beyond breast cancer: A systematic review and meta-analysis of cancer-related secondary lymphedema. Cancer.

[17] Moody J.A., Botham S.J., Dahill K.E., Wallace D.L., Hardwicke J.T. (2017). Complications following completion lymphadenectomy versus therapeutic lymphadenectomy for melanoma—A systematic review of the literature. Eur. J. Surg. Oncol..

[18] Friedman J.F., Sunkara B., Jehnsen J.S., Durham A., Johnson T., Cohen M.S. (2015). Risk factors associated with lymphedema after lymph node dissection in melanoma patients. Am. J. Surg..

[19] Boccardo F., Casabona F., De Cian F., Friedman D., Villa G., Bogliolo S., Ferrero S., Murelli F., Campisi C. (2009). Lymphedema microsurgical preventive healing approach: A new technique for primary prevention of arm lymphedema after mastectomy. Ann. Surg. Oncol..

[20] Dean L.T., Moss S.L., Ransome Y., Frasso-Jaramillo L., Zhang Y., Visvanathan K., Nicholas L.H., Schmitz K.H. (2019). "It still affects our economic situation": Long-term economic burden of breast cancer and lymphedema. Support. Care Cancer.

[21] Bowman C., Piedalue K.A., Baydoun M., Carlson L.E. (2020). The Quality of Life and Psychosocial Implications of Cancer-Related Lower-Extremity Lymphedema: A Systematic Review of the Literature. J. Clin. Med..

[22] Taghian N.R., Miller C.L., Jammallo L.S., O’Toole J., Skolny M.N. (2014). Lymphedema following breast cancer treatment and impact on quality of life: A review. Crit. Rev. Oncol. Hematol..

[23] Sars C., Gillgren P., Schultz I., Lindqvist E.K. (2020). Risk Factors for Complications and Long-Term Outcomes Following Completion Lymph Node Dissection for Cutaneous Melanoma: A Retrospective Cohort Study. J. Plast. Reconstr. Aesthet. Surg..

[24] Gjorup C.A., Dahlstroem K., Hendel H.W., Drzewiecki K.T., Klausen T.W., Holmich L.R. (2021). Factors associated with melanoma-related limb lymphoedema. Acta Oncol..

[25] Jorgensen M.G., Toyserkani N.M., Thomsen J.B., Sorensen J.A. (2018). Surgical-site infection following lymph node excision indicates susceptibility for lymphedema: A retrospective cohort study of malignant melanoma patients. J. Plast. Reconstr. Aesthet. Surg..

[26] Serpell J.W., Carne P.W., Bailey M. (2003). Radical lymph node dissection for melanoma. ANZ J. Surg..

[27] Jorgensen M.G., Toyserkani N.M., Sorensen J.A. (2018). The effect of prophylactic lymphovenous anastomosis and shunts for preventing cancer-related lymphedema: A systematic review and meta-analysis. Microsurgery.

[28] Cakmakoglu C., Kwiecien G.J., Schwarz G.S., Gastman B. (2020). Lymphaticovenous Bypass for Immediate Lymphatic Reconstruction in Locoregional Advanced Melanoma Patients. J. Reconstr. Microsurg..

[29] Chungsiriwattana W., Kongkunnavat N., Kamnerdnakta S., Hayashi A., Tonaree W. (2022). Immediate inguinal lymphaticovenous anastomosis following lymphadenectomy in skin cancer of lower extremities. Asian J. Surg..

[30] Reticker-Flynn N.E., Zhang W., Belk J.A., Basto P.A., Escalante N.K., Pilarowski G.O.W., Bejnood A., Martins M.M., Kenkel J.A., Linde I.L. (2022). Lymph node colonization induces tumor-immune tolerance to promote distant metastasis. Cell.

